# Short-term geriatric assessment units: 30 years later

**DOI:** 10.1186/1471-2318-10-41

**Published:** 2010-06-22

**Authors:** Judith Latour, Paule Lebel, Bernard-Simon Leclerc, Nicole Leduc, Katherine Berg, Aline Bolduc, Marie-Jeanne Kergoat

**Affiliations:** 1 Research Centre, Institut universitaire de gériatrie de Montréal, 4565 Chemin Queen-Mary, Montréal (QC), H3W 1W5, Canada; 2 Service de gériatrie, Hôpital St-Luc, Centre hospitalier de l'Université de Montréal, 1058 rue St-Denis, Montréal (QC), H2X 3J4, Canada; 3 Direction de santé publique et d'évaluation, Agence de la santé et des services sociaux de Lanaudière, 245 rue du Curé-Majeau, Joliette (QC), J6E 8S8, Canada (at the time of the study); 4 Department of Health Administration, School of Public Health, Université de Montréal, Pavillon Mont-Royal, 1430 boul. Mont-Royal, Montréal (QC), H2V 4P3, Canada; 5 Department of Physical Therapy, University of Toronto, 160-500 University Avenue, Toronto (ON), M5G 1V7, Canada

## Abstract

**Background:**

The increasing number of hospitalized elderly persons has greatly challenged decision makers to reorganize services so as to meet the needs of this clientele. Established progressively over the last 30 years, the short-term Geriatric Assessment Unit (GAU) is a specialized care program, now implemented in all the general hospital centres in Quebec. Within the scope of a broader reflection upon the appropriate care delivery for elderly patients in our demographic context, there is a need to revisit the role of GAU within the hospital and the continuum of care. The objective of this project is to describe the range of activities offered by Quebec GAU and the resources available to them.

**Methods:**

In 2004, 64 managers of 71 GAU answered a mail questionnaire which included 119 items covering their unit's operation and resources in 2002-2003. The clinical and administrative characteristics of the clientele admitted during this period were obtained from the provincial database Med-Echo. The results were presented according to the geographical location of GAU, their size, their university academic affiliation, the composition of their medical staff, and their clinical care profile.

**Results:**

Overall, GAU programs admitted 9% of all patients aged 65 years and older in the surveyed year. GAU patients presented one or more geriatric syndromes, including dementia. Based on their clientele, three distinct clinical care profiles of GAU were identified. Only 19% of GAU were focused on geriatric assessment and acute care management; 23% mainly offered rehabilitation care, and the others offered a mix of both types. Thus, there was a significant heterogeneity in GAU's operation.

**Conclusions:**

The GAU is at the cutting edge of geriatric services in hospital centres. Given the scarcity of these resources, it would be appropriate to better target the clientele that may benefit from them. Standardizing and promoting GAU's primary role in acute care must be reinforced. In order to meet the needs of the frail elderly not admitted in GAU, alternative care models centered on prevention of functional decline must be applied throughout all hospital wards.

## Background

In several countries various forms of specialized geriatric units and approaches to adapted care have been established to meet the growing pressure from the number of hospitalized elderly [1-13]. For example, in a Geriatric Evaluation and Management Unit (GEMU) [3,7,11,14-16] the patient is most often transferred from other wards for the sub-acute phase of her/his illness and admitted under the responsibility of the geriatric team, whereas, in the Acute Geriatric Unit [13] the geriatric team is most often responsible for the acute care. In the Acute Care of Elders Unit [5,17,18], the physical surroundings are adapted, a preventive and interdisciplinary approach is applied, without, however, the geriatric team being the sole treatment team. In general, these units characteristically admit a frail population with at least one inability to perform the activities of daily living (ADL), but that must also present the potential to benefit from the intervention. The patients undergo an interdisciplinary assessment and a centered patient care plan in order to minimize, among other things, iatrogenia. Early discharge planning is the rule and pertinent patient information is transmitted to the primary care team.

The province of Quebec has close to 7 million inhabitants, of which 14% are aged 65 years or older [19]. The elderly alone mobilize one-half of hospitalization days [20]. The increase in admissions and beds occupied is proportionally more marked in the 75 years and older group. When admitted to hospital, this clientele more often presents an atypical clinical profile, functional decline, and multiple co-morbidities.

Quebec's population enjoys a universal health care insurance plan. Support home care, assessment, and rehabilitation for frail elderly patients are provided by the Health and Social Services Centres of Quebec: an administrative institution combining local community service centres, as well as long-term and rehabilitation care centres, and a general hospital that assumes responsibility for a population in a given territory. Inspired by the British model [21], GAU were initiated in university affiliated hospitals at the end of the seventies. They were not officially integrated with the other existing geriatric programs (day hospitals, geriatric rehabilitation units, etc.). This specialized care program was originally created to offer to the frail elderly hospitalized with acute conditions, global and integrated health care in an adapted physical environment, and to ensure a comprehensive assessment and intervention by a multi-professional team [22-24]. Until 1999, GAU were progressively established in all general hospitals with a partial goal of alleviating the congestion of the emergency room by elderly patients.

Health ministry policy-makers and hospital administrators joined to make major changes in the organisation of hospital services to better meet the needs of this growing number of elderly and vulnerable patients [25]. From this perspective, a thorough evaluation of the structure and process of the GAU program is deemed pertinent after 30 years of existence. Since these programs have multiplied in number throughout Quebec, we would expect to observe a great diversity both at the level of the resources used in each unit and clinical activities offered. This variety could underline and expose elderly unmet needs.

The main objectives of this article are to describe the operation of the Quebec GAU and their resources and to discuss their specific role in face of the increasing needs of the vulnerable elderly.

## Methods

### Identification of GAU

In 2004, 71 GAU were identified after consulting with the directors of professional services of the 80 general hospitals within Quebec. The GAU are found in regions covering 98% of the total population of the territory.

### Source of Data

#### Operation and Resources of the GAU

A questionnaire was developed to collect information on the operation and resources of the GAU between April 1, 2002 and March 31, 2003. It was elaborated from a critical review of the literature on environmental and organizational factors that can influence the quality of care [26-30], and from recommendations by two experts on health care management. It was then pretested by five GAU managers. Technical improvements (page design, precision of terms) were then made. The final computerized version of the questionnaire consists of 119 questions on the following aspects: administrative structure, patient's medical conditions and clinical stability, physical environment, access to diagnostic services and to other geriatric programs, admission procedures and length of stay, assessment of and intervention in clientele, planning of discharge and follow-up, quality-control procedures, general activities, as well as human and material resources. The respondents also had to rate, on a scale of 0 to 10, their degree of satisfaction with the areas evaluated. Satisfaction was determined by a rating of 7 out of 10 and higher. They were also asked to comment and give reasons for dissatisfaction. The questionnaire was carried out by mail. Of the 71 GAU, the managers (head nurse and physician) of 64 (90%) accepted to participate in the study.

#### Hospitalizations in GAU

The clinical and administrative data pertaining to patients in Quebec hospitals and, more specifically in the GAU, between April 1, 2002 and March 31, 2003 were extracted from the provincial hospital discharge database Med-Echo and were obtained directly from the Ministère de la santé et des services sociaux du Québec (MSSS) [31]. They include socio-demographic characteristics of the patients (age, sex, civil status, living arrangements), type of admission, orientation at discharge, care provided and hospital services concerned, as well as the primary and secondary diagnoses that led to hospitalization based on the International Classification of Diseases (ninth Revision). The clinical severity and risk of mortality indexes [32], based on the classification system "All Patient Refined Diagnosis Related Groups (APR-DRG)", associated with each hospitalization were also extracted from the Med-Echo database. The index of clinical severity indicates the presence of significant interactive factors, co-morbidities, or complications (degree of physiological decompensation) which affect the intensity of services required for patient care. The index of risk of mortality indicates the risk of death in relation to a grouping of hospitalizations considered to be similar clinically and in terms of the volume of resource utilization. The volume of admissions to GAU was determined by calculating the number of hospitalizations per bed in the unit. In the case of four of the 64 GAU the data listed above could not be extracted from the Med-Echo database as they were combined with general medical data or were not available for the period under study.

### Statistical Analyses

A descriptive analysis was carried out using SPSS^® ^software version 14.0. Results were presented according to the geographical location of GAU, their size (number of beds), their university academic affiliation, the composition of their medical staff, and their clinical care profile. The variables were described by proportions and means (or median values if not normally distributed). Student's *t *test, one-way analysis of variance and when needed Mann-Whitney *U *test and the Kruskal-Wallis test were used to detect differences between the categories of GAU. Chi-square test was used for categorical variables. Post hoc multiple comparison adjustments were made using the Tukey or Tamhane methods.

A typology of clinical profiles of GAU was derived from data about patient characteristics and required care at admission, using a two-step cluster analysis procedure. Selected variables were: 1) percentage of medically unstable patients admitted from the emergency department; 2) percentage of patients requiring assessment and acute care while preserving their usual functional capabilities during this period; 3) percentage of patients with a condition where diagnoses were well identified and health status stabilised but requiring intensive rehabilitation treatment (more than three weeks of professional intervention). We first performed a Ward's hierarchical agglomerative cluster analysis using squared Euclidean distance in order to determine the appropriate number of clusters in the sample. We next conducted a k-means iterative partitioning analysis to update cluster centers iteratively and to assign cases to the cluster groups.

### Ethical approval

The study was approved by the Medical Director and the Research Ethics Committee of the Institut universitaire de gériatrie de Montréal, as well as by all Medical Directors in the participating hospitals, and by the Research Ethics Committees of nine hospitals that had required a separate evaluation.

## Results

### Number of Beds and Clinical Profile of Care

The average size of a GAU was 15 beds but the range varied from 4 to 40 beds (Table 1). Overall, the 71 GAU inventoried in all of Quebec operate 1,071 beds (972 in the 64 participating GAU). For the year 2003, this corresponds to a ratio of 10.6 beds in GAU for every 10,000 persons, aged 65 years and older [33]. Half the GAU have 15 beds or fewer, located in hospitals that average a total of 230 beds. For their part, the GAU with more than 15 beds are located in hospitals that average 460 beds. Thirty-six percent of GAU are located in central, urbanized regions and they alone admit 55% of all elderly persons hospitalized in GAU (Table 1). Close to one-third of the GAU are part of a university affiliated hospital; they treat more than 50% of the admissions to the GAU (Table 1). The large majority of GAU (80%) have general practitioners as physicians; 11% are staffed exclusively by geriatricians; in 9% the medical team includes physicians from both disciplines (Table 1).

**Table 1:** Distribution of GAU and hospitalizations according to five groupings


**Groupings**	**GAU** **(*n *= 64)**	**Hospitalizations** **(*n *= 15 575)**

	**%**	**%**

Regions^1^		
Central	36	55
Peripheral to central	23	24
Intermediate	30	18
Distant	11	3
University affiliation		
Yes	30	53
No	70	47
Clinical care profile		
Acute care/assessment	19	34
Mixed care	58	54
Rehabilitation	23	12
Size (number of beds)		
4-9	31	10
10-15	23	17
16-20	22	32
21-40	23	41
Composition of medical staff		
General practitioners	80	67
General practitioners and geriatricians	9	15
Geriatricians	11	18


^1 ^Central regions = Montréal, Québec, and Laval; regions peripheral to central = Chaudière-Appalaches, Lanaudière, Laurentides et Montérégie; intermediate regions = Bas-Saint-Laurent, Saguenay-Lac-St-Jean, Mauricie-Bois-Franc, Estrie et Outaouais; and distant regions = Abitibi-Témiscamingue and Gaspésie-Îles-de-la-Madeleine.

The cluster statistical procedure revealed the presence of three distinct GAU clinical profiles of care: GAU providing mainly assessment and care of acute conditions; GAU oriented towards intensive rehabilitation; and mixed-type GAU providing assessment and acute care, as well as non-intensive functional rehabilitation interventions. Fifty-eight percent of the GAU have a mixed care profile (assessment/acute care and rehabilitation) and deal with approximately half the hospitalizations in GAU (Table 1). Only 19% of the GAU were focused mainly on assessment and acute care (Table 1).

### Clientele

In the period under study, of all the hospitalizations of any kind of persons aged 65 years and older (*n *= 166,232) that occurred in hospitals, 9% were admitted in GAU (*n *= 15,575). Table 2 presents the characteristics of the entire clientele of the GAU and the extent of variations among them. The average age of the patients hospitalized is 81 years; 67% are women and 37% are married. They have been admitted mainly through transfer (53%) from another department of the same hospital or directly from the emergency department (34%). The average length of stay in GAU is 23 days (median of 16), four of which, on average, are due to waiting for a long-term care facility; and 33 days in total (median of 23). The average length of stay in GAU is significantly higher (*p *≤ 0.001) in ones that have a clinical care profile of rehabilitation (34 days) than in those that are of mixed type (22 days) and for assessment/acute care (21 days). The length of stay in a GAU is shorter at non-university affiliated GAU (21 vs. 25 days, *p *≤ 0.001); however, the average total length of hospital stay is shorter in university affiliated GAU (30 vs. 37 days, *p *≤ 0.001).

**Table 2:** Characteristics of clientele hospitalized in GAU

Variables	**For all GAU hospitalizations in Quebec**^**1**^ (*n *= 15 575)	**By individual GAU**^**1**^ **(*n *= 60**^**2**^**)**
	**Mean ± standard deviation**	**Minimum and maximum mean**

Age in years	81 ± 8	74 - 84
Length of stay in geriatric ward (days)	23 ± 28	12 - 64
Length of stay in hospital (days)	33 ± 36	14 - 85
Index of clinical severity	2.7 ± 0.8	1.9 - 3.2
Index of risk of mortality	2.4 ± 0.8	1.6 - 2.8
Number of secondary diagnoses	10 ± 4	4 - 14

	%	Minimum and maximum mean percentages

Sex		
Female	67	48 - 76
Civil status		
Single	16	3 - 43
Married	37	19 - 61
Widow (er) Separated Divorced	47	16 - 64
Type of admission		
Emergency department	34	0 - 100
Intra-hospital transfer	53	0 - 100
Inter-hospital transfer	7	0 - 88
Home (specific)	7	0 - 58
Orientation at discharge		
Home	67	44 - 88
Hospital or rehabilitation centre	5	0.3 - 16
Long-term care facility	20	5 - 41
Death	8	2 - 28
Principal diagnoses		
Mental disorders (including dementia)	18	2 - 66
Illnesses of the circulatory system	16	3 - 29
Traumatic injuries and poisonings	12	1 - 34
Ill defined symptoms, signs, and morbid states	12	1 - 95
Illnesses of osteo-articular system, of muscles, and of connective tissue	10	1 - 26


^1 ^The sampling unit in the first column is the individualized hospitalizations in a GAU in Quebec, whereas the sampling unit in the second column is the individualized mean GAU (i.e. the mean of the individualized hospitalizations for each GAU).

^2 ^The data are missing for four GAU.

Upon discharge from the GAU, 67% of patients return to live in their home or in a community residence; 20% are redirected to long-term care facilities; 5% are transferred to a rehabilitation centre; and 8% died during hospitalization. The main diagnoses most frequently identified during hospitalization have to do with mental disorders, including dementia (18%) and, diseases of the cardiovascular system (16%). The number of secondary diagnoses is, on average, 10 per patient.

The patients in the GAU of the central regions differ from those of the other regions (*p *≤ 0.05): they are older (82 vs. 78-80 years old) and are admitted in greater proportion through the emergency department (52% vs. 18-34%); their indexes of clinical severity and risk of mortality are slightly higher (2.72 vs. 2.64-2.70 and 2.38 vs. 2.28-2.34 respectively); their main diagnosis is more often a mental disorder, including dementia (21% vs. 8-15%); and they are more often directed to a long-term care centre upon discharge (23% vs. 15-19%). Significantly higher values are also observed in the GAU located in university affiliated hospitals, in large hospitals that have as their clinical profile assessment/acute care, and in the GAU where only geriatricians practice.

### Number of Admissions

The number of admissions to GAU per bed is, on average, 16 and varies between 4 and 56. This ratio is significantly lower for the GAU that have a clinical care profile of rehabilitation (10), in comparison with the GAU that have a profile of assessment and acute care (24) (*p *≤ 0.05) or of mixed type (16) (*p *≤ 0.01). More admissions are observed in GAU affiliated with a university (20 vs.14, *p *= 0.06).

### Physical Environment

The majority of GAU (70%) share their physical environment with another department. In 75% of cases, they have the benefit of the premises used for rehabilitation intervention (physiotherapy, occupational therapy), located on the same floor as the program. There was an absence of an electronic tracking system for wandering and at risk of getting lost patients in 42% of the GAU and of a common dining room in 33%. In addition, 30% of GAU reported environmental constraints including physical and structural limitations for common physical disabilities.

### Admission Procedures and Criteria

In most GAU (74%) the physician decides admission, however, this proportion is lower within non-university affiliated GAU (64% vs. 95%, *p *≤ 0.05). In the other cases (26%), this task devolves to an admission committee, composed of health care professionals or clinical managers. The following main exclusion criteria were common: need for palliative care (67%) or permanent institutionalization (49%) or convalescence (46%); suicidal patients (46%) or those admitted principally because they suffer from a psychiatric pathology (40%); or patients who require temporary institutionalization for social or medical reasons (37%). Compared to university affiliated GAU, the non-university affiliated GAU exclude in a larger proportion: patients who are not ambulatory (30% vs. none, *p *≤ 0.01), those having a psychiatric pathology as principal diagnosis (51% vs. 11%, *p *≤ 0.01), needing palliative care (84% vs. 22%, *p *≤ 0.001) or a temporary placement for social or medical reasons (44% vs. 17%, *p *≤ 0.05).

### Number and Characteristics of Health Care Professionals

#### Medical staff

The medical responsibility for the patients is taken on mostly by the staff physician in the GAU (80%) or it is shared with the patient's family doctor (20%). For purposes of comparison to a medium-sized GAU, we find, if we calculate the number of staff per 15 beds, that the number of physicians covering the GAU during the day in a typical week is, on average, 2.4 per 15 beds. This ratio is higher (3.8) within a GAU having 4-9 beds than in other categories of GAU according to size (10-15 beds: 2.3; 16-20 beds: 1.9; 21-40 beds: 1.5, *p *≤ 0.001). Also, it was lower in university affiliated GAU (1.7 vs.2.9, *p *≤ 0.01). Depending on the composition of the medical staff, the number is 1.0 for the GAU having exclusively geriatricians; 1.7 for those having general practitioners and geriatricians; and 2.7 for those exclusively having general practitioners. Most physicians practice other clinical or administrative activities in other departments or outside the hospital. In fact, physicians working exclusively in GAU are found in only 6% of the units. In 71% of the GAU, the physicians work four consecutive weeks or longer in the units, and this proportion is higher in university affiliated GAU (95% vs. 61%, *p *≤ 0.01). Close to 60% of the on-call periods in GAU are assumed exclusively by the physicians of the unit.

#### Other Health Care Professionals

The ratio in the median number of physiotherapists and occupational therapists drops significantly according to the increase in the size of GAU (Table 3). It is equally lower per 15 beds (*p *≤ 0.05) in the GAU of the central regions (physiotherapists, 0.78 vs. 1.10-1.50; occupational therapists, 0.57 vs. 1.10-1.28); in those affiliated with universities (physiotherapists and occupational therapists, 0.75 vs. 1.15); and in those whose clinical care profile focuses mainly on assessment/acute care (physiotherapist, 0.75 vs. 1.0 (mixed care) vs. 1.2 (rehabilitation)). Generally speaking, we observe a constant ratio in the total number of nursing staff per bed (head nurse, assistant head nurse, registered nurse, assistant nurse, attendant), depending on the various categories of GAU. In all, their median value is 4.8, 2.8, and 1.8 per 15 beds during the day, evening, and night shifts respectively. All the GAU have occupational therapists and 97% have physiotherapists (the others have physical technicians), while social workers, dietitians, pharmacists, and liaison nurses are found in 89%, 87%, 66%, and 45% of GAU respectively (Table 3). A liaison nurse is considered separate to nursing staff in that his/her role is mainly one of "liaison" with community health professionals and families.

**Table 3:** Ratios of health care professionals according to the size of the GAU


**Health Care Professionals**	**GAU**				
	**Total** **(*n *= 62**^**1**^**)**	**4-9 beds** **(*n *= 19)**	**10-15 beds** **(*n *= 14)**	**16-20 beds** **(*n *= 14)**	**21-40 beds** **(*n *= 15)**

	Median (25^th ^and 75^th ^percentiles) of equivalent number of full time/15 beds				
Physiotherapist	1.0 (0.8 - 1.3)	1.5**	1.2*	0.9	0.7
Occupational therapist	1.0 (0.8 - 1.3)	1.5**	1.1	0.9	0.7
Social worker	0.9 (0.6 - 1.2)	0.8	1.1	0.9	0.8
Dietitian	0.3 (0.2 - 0.6)	0.4	0.4	0.2	0.3
Pharmacist	0.3 (0.1 - 0.5)	0.2	0.2	0.4	0.2
Liaison nurse	0.6 (0.1 - 0.5)	0.9	0.4	0.2	0.5

* Significant difference with GAU having 21-40 beds (*p *≤ 0.001)

** Significant difference with other three categories taken one by one (*p *≤ 0.05)

^1 ^The data are missing for two GAU.

### Activities of Continuing Professional Education

During the period under study, an activity of professional education in geriatrics/gerontology was offered to the entire multi-professional team in 44% of the GAU and to health care professional groups in 38% of the units. The nurse and the physician in charge of the GAU benefited from professional development activities in management in 39% and 13% of GAU respectively. The university affiliated GAU offer a higher proportion of continuing management training to head-nursing staff (58% vs. 31%, *p *≤ 0.05) and continuing clinical training to the multi-professional team (63% vs. 36%, *p *≤ 0.05).

### Assessment and Intervention Procedures

The assessments done by the health care professionals are more often systematic, rather than following medical consultation. This is respectively the case in 73%, 72%, 63%, 56%, and 51% of the GAU for physiotherapists, social workers, occupational therapists, dietitians, and pharmacists. At admission, a systematic evaluation of patients by physiotherapists (83% vs. 53%, *p *≤ 0.05) and occupational therapists (72% vs. 42%, *p *≤ 0.05) is more frequently done in non-university affiliated GAU.

A multi-professional meeting takes place at least once a week in all GAU. It is generally led by a member of the nursing staff (50%) or by a physician (33%). In the GAU where these health care professionals are available, the presence at this meeting of a pharmacist (81%) or a liaison nurse (68%) is less common than that of the physician, nurse, occupational therapist, physiotherapist, social worker, and dietitian (89-100%). According to the managers, plans for inter-professional intervention are executed in 95% of the GAU, but only 23% are archived after hospitalization. There was no statistically significant difference in the archiving habits within the different GAU groups studied.

### Procedures for Discharge Planning

Clinical information about the patient is generally collected by medical or nursing staff in the 48 hours following their entry into the program. In 96% of the GAU, it is obtained from the family or close relatives; in 42%, from the first line services; in 18%, from the family doctor.

GAU managers (98%) state that meetings are held systematically with the patient and his/her relatives to communicate the results of hospitalization and to transmit recommendations. As for the planned follow-up of the patients who return to their home, only 23% of the GAU offer post-hospitalization follow-up services in the form of a telephone reminder or a visit to an outpatient clinic.

The medical discharge summary is the document most often transmitted by the GAU to the family doctor (41%). It is more frequently transmitted by university affiliated GAU (63% vs. 32%, *p *≤ 0.05) and those having 16-20 beds (71% vs. 4-9 beds: 20%; 10-15 beds: 40%; 21-40 beds: 43%, *p *≤ 0.05). Furthermore, an inter-institutional form, containing the diagnoses, the main recommendations for nursing care, and the list of medications, is transmitted to the homecare service of the CLSC (Local community health care and service centre), preceded by a telephone communication, by 56% of the GAU.

### Access to Other Services and Geriatric Programs

Overall, the day care programs are the most widespread, available to 92% of the hospitals. GAU have priority in only 33% of the hospitals. The day hospital programs are the least available (56%), with prioritized access to the GAU in approximately 40% of the hospitals. As for the functional and intensive rehabilitation units, they are generally available (84%), but priority access is given to the GAU in fewer than half the hospitals (46%). As for the ambulatory services in 2002-2003, 33 of the 64 hospitals had outpatient clinics dedicated exclusively to geriatrics. These clinics include outpatient clinics for geriatrics (42%), geronto-psychiatry (27%), functional assessment (19%), cognition (7%), continence (3%), and chronic pain management (2%). Day hospitals (90% vs. 42%, *p *≤ 0.001), geriatric out-patient clinics (63% vs. 29%, *p *≤ 0.05), and psycho-geriatric out-patient clinics (58% vs. 13%, *p *≤ 0.001) are more available in university affiliated GAU. Day hospitals (93% vs. 30% to 79%, *p *≤ 0.001) and geriatric out-patient clinics are more available within GAU having 21-40 beds (73% vs. 25% to 43%, *p *≤ 0.01).

### Mechanisms to Assess Quality of Care

The screening or preventive protocols that are used in the GAU concern nosocomial infections (81%), pressure ulcers (77%), use of restraints (59%), episodes of constipation (58%), errors in medication administration (53%), falls/fractures (50%), pain (33%), urinary incontinence (27%), immobility (17%), and delirium (11%). The intervention's protocols that are used in the GAU target pressure ulcers (78%), nosocomial infections (78%), errors in medication administration (73%), episodes of constipation (72%), use of restraints (66%), pain (38%), falls/fractures (34%), urinary incontinence (34%), immobility (14%), and delirium (8%). University affiliated GAU have more often elaborated higher number (≥8) of both types of protocols about these ten problem issues (37% vs. 13%, *p *≤ 0.01).

### Level of Satisfaction among Managers of GAU

About half the managers of the GAU (51%) declared themselves to be satisfied (satisfaction rating equal to or higher than 7 on a scale of 10), with the range of programs and geriatric services offered outside the hospital in which the GAU is located; only 35% are satisfied with the waiting times for accessibility. The comments collected addressed a lack of diversity of programs and services offered (rehabilitation, beds for temporary accommodation or transition, beds to tide over, familial-type intermediary resources).

Only 38% of the managers are satisfied with the computer resources available in the GAU; 54% with the allocated budget. The comments collected displayed the lack of information technology positions and of software to manage the patients' clinical data. Fewer than half (43%) of the managers are satisfied with the possibilities of continuous education for the health care professionals of the GAU. The principal barriers for training activities are the lack of a budget and of time on the part of the staff. Approximately one-half or fewer of the managers are satisfied with the number of physicians (54%), pharmacists (52%), and clinical nurses specialized in geriatrics (42%), that are available as core members of the GAU team. In terms of medical resources, there was a shortage of physician specialists (geriatricians, psychiatrists, physiatrists, and neurologists) and of treating physicians with primary assignments to the GAU. The general lack of resources was also reported as a concern relative to other health care professionals; specifically the clinical nurses specialized in geriatrics, the pharmacists and the accessibility to a neuropsychologist.

Fifty-seven percent of the managers are satisfied with the budget allocated for the material resources of the GAU. However, they reported that the material needs specific to geriatrics (geriatric chairs, bladder scans, electric beds, electronic tracking system for people at risk of getting lost, walking equipment) were under-financed. The environment was also a major source of dissatisfaction, with only 61% of the managers being satisfied with the design of the physical premises.

## Discussion

The principal objective of this article was to take stock of the current state of the GAU program in Quebec, 30 years after its implementation. In general, five major conclusions emerge from the observed results.

Firstly, accessibility in regards to the GAU program is limited. In fact, even though the GAU are generally well distributed and established in the hospitals of Quebec, especially in the university affiliated hospitals, the number of beds has decreased since the 2000's, and this, despite the increase number of frail elderly patients admitted in general hospitals [34,33]. What is more, this poor accessibility has not been compensated by being treated as a priority, nor with a better integration of GAU with the other geriatric programs available outside the hospital setting.

Secondly, the client-type admitted to the GAU is generally very old (≥80 years) and often presents cognitive disorders simultaneously associated with one or several geriatric syndromes. These complex health problems require that a multi-professional team, trained in elderly care, assume total responsibility [4,9,14,17,35,36].

Thirdly, a discrepancy was observed between the original mission of the GAU, identified as assessment and acute care of conditions [22], and that which was seen to be practiced in several GAU, where intensive rehabilitation care still predominated. As the average stay in a GAU for assessment and acute care is, all the same, three weeks, we observe that, in general, the patient's return to fitness is ensured on the spot during the episode of care. This follows the British geriatric model of the 1980's-1990's [21] and that of the GEMU of the 1980's [1,16,37]. These various clinical care profiles result in adaptations by the GAU in terms of functioning and resources, as was demonstrated following the different GAU groups compared. In fact, additional analyses reveal the existence of correlations among the groupings of GAU retained for the analysis; for example, between geographical location and university affiliation (-0.61); size (-0.50) and composition of medical staff (-0.38). The GAU of the central regions are more often large in size and located in a hospital centre affiliated with a university, and have, more often than not, geriatricians working there.

Fourthly, the GAU whose clinical care profiles focus on assessment and acute care are overloaded. In fact, their average volume of admission per bed (24) is higher than should be the case (18), in relation to the average length of stay (21 days). This means that many patients who are accepted in these GAU must be treated outside the unit.

Fifthly, the rather low rates of satisfaction reported by the managers of the GAU regarding the shortage of medical and specialized health care professional resources available in the GAU, and the lack of availability and accessibility to the other geriatric resources outside the hospitals, indicates that the managers of the GAU need changes to improve the operation of their units.

The second objective of this article was to discuss the role of the GAU in view of the growing need for care and hospital services for the vulnerable elderly. At the present moment, some still question the efficiency of geriatric hospital care [8,9,11,13,38], while others report that the programs and approaches that are adapted to the elderly show higher clinical benefits than those of the conventional biomedical models [2,5,6,10,12,17,18,39]. The analysis of the situation of the Quebec GAU shows that this type of program plays an essential role in the hospital care given to the vulnerable elderly, whose complex problems require cutting-edge expertise in geriatric care. However, the results obtained show that the program is struggling to fulfill its role in the current system of elderly care. Considering the context of demographic ageing and the limited capacity of the GAU, we propose a number of recommendations regarding the targeting of clientele, the physical environment, the medical and health care professional resources, as well as the clinical and management procedures, and those of discharge planning.

### Targeting of Clientele

We consider that the clinical mission of the GAU should not be that of an intensive rehabilitation program, particularly when this structure already exits in the administrative health region. Moreover, the recognized exclusion criteria of the GAU program [40] should be applied more systematically in the cases of clientele requiring palliative care, convalescence, long-term care, or major psycho-geriatrics. In order to protect its primary mission of assessment and acute care, it will be necessary, on the one hand, to increase the number of alternative resources and complementary programs in the care network and, on the other, to give priority to the GAU for the transfer of its patients to the other departments and geriatric programs, once the acute care episode has been completed.

Moreover, we are aware of the fact that the selection of client-type is partly influenced by institutional priorities (e.g. reduction of overload in emergency department), but, above all, it is linked to the availability and ability of physicians and care teams to assume responsibility for patients who are clinically complex and unstable. As reported in their studies [41,42] and according to our results, some GAU appear to select patients who are medically stable, so as to control the volume of admissions and to maximize the work of the rehabilitation therapists, while others work constantly with a clientele requiring acute care in an overloaded unit and with an insufficient multi-professional team.

In order to improve this situation, it will be necessary, on the one hand, to raise the expertise of all health care professionals in the emergency departments and care units of Quebec hospitals, so that their approach to care is better suited to the needs of the elderly. This will allow both the treatment of acute medical conditions and episodes of decompensation in chronic illnesses, and the prevention of functional decline associated with hospitalization and of any other harmful effects [43]. On the other hand, it will be necessary to improve targeting, by identifying among the elderly and vulnerable clientele admitted to the hospital, those patients that will benefit the most from a global, inter-professional geriatric intervention in GAU, in a context of acute or sub-acute care [40]. It will also be necessary to ensure adequate health care professional resources.

### Physical Environment

The physical premises of the GAU are not always adapted, especially for patients at risk of wandering and getting lost. Moreover, in a very high percentage, there are no nearby premises for rehabilitation treatment or a common room for the purpose of direct observation. An environment, which is ill adapted to the geriatric clientele, can certainly be harmful to the patients' security, and the efficacy and quality of care administered by the health care professionals. It is important to realize that an adapted environment plays a facilitating role in the recuperation process of a hospitalized elderly person and the necessary measures will have to be brought to bear [5].

### Medical and Health care Professional Resources

In general, a medical presence in the GAU ensures that diagnosis and treatment occur for the clientele admitted to these units. However, the medical assumption of responsibility varies among GAU, as to the training of the physicians and their presence in the unit. In 20%-30% of GAU, the physicians work fewer than four consecutive weeks in the unit or in collaboration with the family doctor, given their other clinical or administrative activities, internal or external to the hospital. As has been reported in several studies [17,35,36], the geriatricians are few and are not necessarily part of the team of treating physicians or of consultants. In Quebec, there are actually around fifty certified geriatricians (medical sub-specialty with five-year training in internal and geriatric medicine) in active clinical practice and about the same number of family doctors who hold a certificate attesting to additional training of 6-12 months in elderly care. This gives a relative maximum availability of one physician with expertise in geriatric care per 10,000 elderly, aged 65 years and older, in Quebec. The number of more specialized staff is, therefore, definitely not enough to ensure access to their expertise in all the GAU. However, integrated medical practice centered on the patient, as is practiced by the geriatricians, can be applied by motivated family physicians pursuing continuing education. The creation and establishment of inter-professional protocols, of systematic approaches to geriatric syndromes, and their updating, should become a priority of geriatric services, in order to ensure quality care. Furthermore, the training of new geriatricians and family physicians that have completed additional training in elderly care should also become a priority of the medical faculties and the College of Physicians.

The GAU are generally provided with a complete multi-professional team (physiotherapist, occupational therapist, social worker and/or liaison nurse, dietitian, and pharmacist) able to help globally and efficiently a clientele with complex needs. However, we have observed that their numbers are not always sufficient, particularly in the larger GAU. We also deplore the lack of continuing education activities, both clinical and administrative, in most of the GAU, despite the necessity of maintaining health care professional expertise. In addition to the local supervision of the students who come from various professions, the health care professionals of the GAU will also be called upon more and more to disseminate their expertise within and beyond their hospital centres, to fill the need for training in elderly care within the health network [44-46].

### Procedures of Assessment, Intervention, Planning of Discharge, Evaluation and Improvement of Quality of Care

The results of this inquiry into the GAU lead us to deplore the lack of systematic protocols of assessment, of intervention, and of mechanisms to evaluate and improve the quality of care specific to geriatric syndromes. The same holds true for procedures in planning a discharge and transmitting information upon discharge. Continuing the care together with the primary care team is essential and the summary of the file is a recognized method of transmitting information when the content is brief, pertinent, and structured, and is sent quickly following hospitalization [47-51]. As mentioned earlier but from a different perspective, in order to operate efficiently, the GAU must be able to refer their patients, if necessary, to complementary geriatric services, after their stay in the unit.

### Strengths and Limitations of the Study

The thorough analysis of the structure and processes in most GAU is the principal strength of this study. Only seven of the 71 GAU listed in Quebec (10%) did not participate in the study. They hold between 2 and 35 beds and they originate from different regions. In all, they represent 9% of the beds of the GAU. The reason for refusal to participate was the lack of time on the part of their manpower to answer the questionnaire. Besides the possibility of a biased selection associated with volunteering, that is characteristic of all studies of this nature, everything leads us to consider that the study provides a representative description of the operation and resources of the GAU throughout Quebec.

Nonetheless, although we developed a standardized questionnaire and we invited the managers of the GAU to answer it by consulting their health care professional teams, there was room for subjective interpretation by the manager that could not be verified through the governmental database.

We attempted to make the comparison of the structure and process between some GAU groups. Unfortunately, more often than not, we observed a trend without there being a clear statistical difference. This was because the study lacked sufficient statistical power (type II error). As well, the study was composed of a multitude of comparison tests without an adjustment of the alpha (type I) error. Consequently, it is possible that we rejected the null hypothesis more often than permitted.

## Conclusion and Perspectives

A unit of specialized geriatric care is available in most general and specialized hospital centres in Quebec. However, the small number of physicians devoted to those units, coupled with the growing number of elderly and vulnerable patients, prompts us to favor, as of this moment, a systematic and preventive approach to all elderly (75 years of age and older) admitted to hospital. The targeting of clientele, central to the efficacy of the GAU, must allow the identification within the elderly and vulnerable population admitted to hospital, those patients who will benefit from a global inter-professional geriatric assessment in a GAU, in a context of acute and sub-acute care. There is also a need for greater standardization in the treatment and management of patients in the GAU and better continuity of care upon discharge. The GAU are called upon to contribute to the dissemination of expertise in geriatric care. To optimize outcomes and act as roles models, staff working there must be of sufficient numbers, must have the benefit of continuing education, and must work in a physical environment suited to the needs of the geriatric clientele.

The results and recommendations drawn from this study should be useful in other jurisdictions. This is especially true where the geriatric services are available to elders living in the community throughout an integrated local service network.

## Competing interests

The authors declare that they have no competing interests.

## Authors' contributions

MJK directed and supervised all steps of the study. JL, MJK, PL, AB and BSL drafted the manuscript. AB performed the statistical analysis supervised by BSL. All the authors participated in the design and execution of the study. All the authors reviewed and approved the manuscript.

## Pre-publication history

The pre-publication history for this paper can be accessed here:

http://www.biomedcentral.com/1471-2318/10/41/prepub
